# **Expanding Medical Education and Task Shifting** Comment on "Doctor Retention: A Cross-sectional Study of How Ireland Has Been Losing the Battle"

**DOI:** 10.34172/ijhpm.2020.218

**Published:** 2020-11-08

**Authors:** Daniel R. Arnold

**Affiliations:** Division of Health Policy and Management, School of Public Health, University of California, Berkeley, Berkeley, CA, USA.

**Keywords:** Doctor Retention, Medical Education, Task Shifting, Ireland

## Abstract

Brugha et al provide convincing evidence that Ireland stills need to overcome many hurdles, including poor training and working experiences in Irish hospitals, before it can significantly improve its record on doctor retention. The findings reported by Brugha et al are particularly disappointing in light of the fact that Ireland implemented a doctor retention strategy in early 2015. Ultimately, doctor retention is important because it can help alleviate the health workforce shortages that many countries face currently and that are projected to worsen over the next decade. The purpose of this commentary is to highlight two additional strategies for alleviating health workforce shortages – expanding medical education and task shifting.

## Introduction

Brugha et al nicely lay out the difficulties of doctor retention, both in Ireland specifically and more generally.^1^ In the case of Ireland, the authors provide convincing evidence that many hurdles, including poor training and working experiences in Irish hospitals, still need to be overcome before the country can significantly improve its record on doctor retention. The findings reported by Brugha et al are particularly disappointing in light of the fact that Ireland implemented a doctor retention strategy in early 2015. The authors’ ultimate recommendation that Ireland “needs a more diversified retention strategy that addresses under-staffing, facilitates circular migration by younger trainees who choose to train abroad, identifies and addresses specialty-specific factors, and builds mentoring linkages between trainees and senior specialists” is a good one that makes a great deal of sense based on the evidence provided in the paper.^1^

Ultimately, doctor retention is important because it can help alleviate the health workforce shortages that many countries currently face and which are projected to worsen over the next decade.^2,3^ The purpose of this commentary is to highlight two other ways to alleviate health workforce shortages – expanding medical education and task shifting.

## Medical Education

Of the 1148 junior doctors surveyed by Brugha et al, 35% planned to leave Ireland but return later while 17% planned to leave and not return. In 2018, Ireland had 1224 medical graduates.^4^ Let’s assume that all 17% that plan to leave and not return do so and half of the group that says it plans to return ends up never returning. This implies a retention rate of 65.5% (= 100-17-(35/2)). A 65.5% retention rate would mean only 802 of the 1224 medical graduates in 2018 will ultimately practice in Ireland. If Ireland were to succeed in increasing its retention rate to 90%, 1102 of 1224 graduates in 2018 would ultimately practice in Ireland – 300 more doctors than in the 65.5% retention rate scenario. An increase of 300 doctors is significant for Ireland given it has 15962 practicing physicians in total as of 2018.^4^

**Figure 1:**
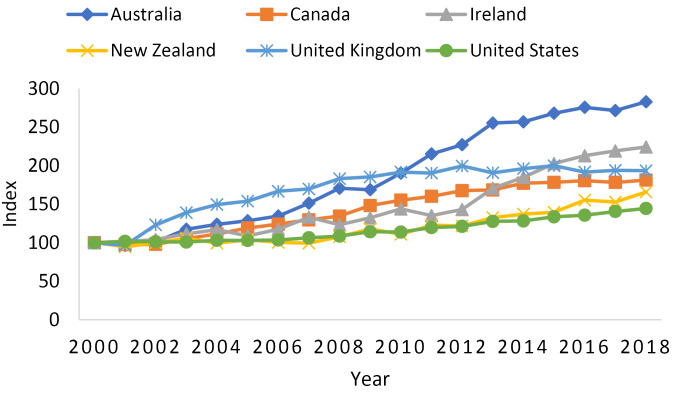
Growth in Medical Graduates for Selected Counties, 2000-2018.

Source: Author’s analysis of data from OECD.Stat.^4^

Notes: Index is calculated as the number of as the number of medical graduates in country *i* in year *t* divided by the number of medical graduates in country *i* in year 2000 (the first year of the study period) multiplied by 100.

The purpose of this thought exercise is to give the reader a sense of how many more doctors would practice in Ireland if doctor retention were improved dramatically. Three hundred additional doctors per year is a substantial increase. But how does this number compare to what would be possible were the number of medical graduates in Ireland to increase substantially per year? Suppose the number of medical graduates in Ireland doubled to 2448. At the originally assumed 65.5% retention rate, this would mean 1604 graduates would ultimately practice in Ireland, or stated another way, 802 more doctors per year than the current situation. This increase dwarfs the 300 increase from improved doctor retention. A doubling in the number of students that medical education can handle might seem optimistic, but it is exactly what Ireland was able to accomplish between 2000 and 2018 (Figure 1). And despite the fact that Ireland has more medical graduates per 100000 population than the Organisation for Economic Co-operation and Development (OECD) average (as noted by Brugha et al), there is still room for improvement; which is generally the case for most countries given the significant global doctor shortages that are forecasted for the coming decade.^2^

Figure 1 plots the growth in the number of medical graduates for six countries – Australia, Canada, Ireland, New Zealand, the United Kingdom, and the United States. To easily compare the growth in medical graduates across countries, I calculated an index as the number of medical graduates in country *i* in year *t *divided by the number of medical graduates in country *i* in year 2000 (the first year of the study period) multiplied by 100. The growth in medical graduates from 2000 to 2018 can then be calculated by subtracting 100 from the value of the index in 2018. For example, Ireland’s index in 2018 was 224, which means the number of medical graduates grew by 124% (ie, more than doubled) between 2000 to 2018. The importance of Ireland continuing its success in graduating more doctors every year should not be lost while discussing doctor retention. Ultimately, improving both doctor retention and the number of medical graduates per year will best enable Ireland to stave off possible doctor shortages in the future.

As a last note on Figure 1, the other five countries plotted were chosen to match the survey results in Brugha et al.^1^ Specifically, Brugha et al asked the junior hospital doctors that intended to leave Ireland which country they intended to migrate to. The authors found 27% intended to migrate to the United Kingdom, 23% to Australia, 22% to Canada, 9% to the United States, 8% to New Zealand, and 11% answered another country. The five countries most commonly mentioned are the ones plotted along with Ireland in Figure 1. It is notable that among these countries, only Australia had a greater percentage increase in the number of medical graduates than Ireland between 2000 and 2018. As increasing the number of medical graduates is one of the key strategies that countries can use to stave off doctor shortages, its troublesome that some of the countries that pull graduates from Ireland the most have had limited success in increasing their number of medical graduates. If these trends continue, it is likely these countries will face greater doctor shortages in the years to come, and be more desperate to lure graduates away from other countries, including Ireland.

The survey evidence in Brugha et al suggests that expanding medical education for women might be a particularly good strategy. The authors note that “females were more likely than males to remain in Ireland (48% vs. 41%) and half as likely to stay abroad (11% vs. 24%).”^1^ Improving the recruitment of women into medical education would ameliorate the flow of trained doctors out of Ireland even in expanding medical education proves financially infeasible.

## Task Shifting

Task shifting is another strategy for dealing with doctor shortages. Task shifting is defined as delegating tasks to existing or new groups of health professionals that have either less training or more narrowly tailored training. For example, in the United States there has been substantial discussion of expanding the responsibilities of nurse practitioners to help with the increase in demand that was generated by recent healthcare reform that increased the number of Americans with health insurance coverage.^5^ A recent review article found considerable evidence that task shifting can help alleviate workforce shortages and skill mix imbalances.^6^

**Figure 2:**
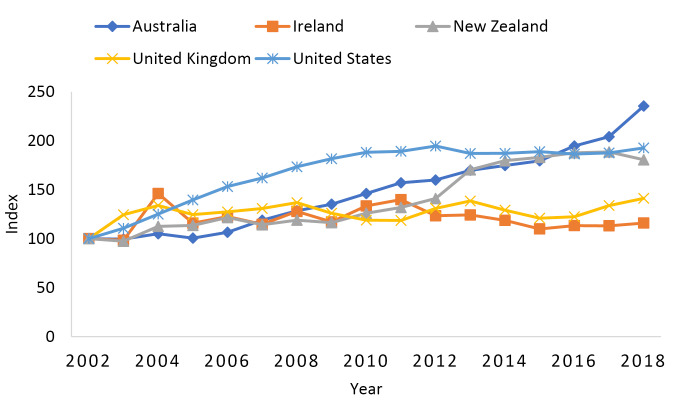
Growth in Nursing Graduates for Selected Counties, 2000-2018.

Source: Author’s analysis of data from OECD.Stat.^4^

Notes: Index is calculated as the number of as the number of Nursing graduates in country *i* in year *t* divided by the number of Nursing graduates in country *i* in year 2000 (the first year of the study period) multiplied by 100.

Maximizing the benefits of task shifting may be where Ireland is coming up short. Figure 2 plots nearly the same information as Figure 1, but for nursing graduates instead of medical graduates. The two differences are that the study period now covers 2002 to 2018 and Canada is excluded due to missing data. While Ireland had the second largest growth in medical graduates behind only Australia (Figure 1), it had the smallest growth in nursing graduates among the five countries plotted in Figure 2. Specifically, the number of nursing graduates in Ireland only increased by 16% from 2002 to 2018. Figure 2 also provides suggestive evidence that the United States is undertaking task shifting. In Figure 2, growth in the number of nursing graduates was second behind Australia at 93% between 2002 and 2018. Back in Figure 1, the United States had the lowest growth in the number of medical graduates between 2000 and 2018 at 44%.

Assuming the quality of healthcare services is able to remain high when tasks are shifted from doctors to health professionals with less training, task shifting has three major benefits. First, health workforce shortages can be more quickly alleviated because the time it takes to train non-doctor healthcare workers is often substantially less than the time it takes to train doctors. Second, the cost to the health system is generally reduced as doctor salaries are often significantly higher than the salaries of non-doctor healthcare professionals. Third, doctors generally do not like the “non-core task” aspects of their jobs. According to the Brugha et al survey results, half of the doctors who planned to quite medicine cited non-core tasks as factor in their decision to leave the profession.^1^ This leads one to believe that shifting non-core tasks to non-doctor healthcare professionals would improve doctor retention.

## Conclusion

Based on current trends, the health workforce shortages that many countries are already experiencing are likely to be exacerbated in the decade to come.^2,3^ Improving doctor retention – just like expanding medical education and task shifting – is a way to alleviate health workforce shortages. Which strategy (or combination of strategies) a country chooses to undertake will depend on its level of available resources and political will. Implementing the recommendations of Brugha et al for improving doctor retention would be much less resource intensive than expanding the capacity of medical education. The resource intensity of task shifting probably follows somewhere in between. There are some services that non-doctor healthcare professionals are capable of performing today without any additional training. There are other services that would also likely benefit from task shifting, but that might require some additional training of non-doctor health professionals before the tasks can be safely shifted. Ultimately, a combination of these strategies (and others) will likely be necessary if countries are to avoid the dramatic health workforce shortages that are projected for the next decade.

## Ethical issues

 Not applicable.

## Competing interests

 Author declares that he has no competing interests.

## Author’s contribution

 DRA is the single author of the paper.
